# Wearable Dual-Mode Biosensing System for Dynamic Light Dosimetry in Tissues

**DOI:** 10.3390/bios16050263

**Published:** 2026-05-02

**Authors:** Jun Wei, Lansixu Ma, Wenxuan Li, Peng Xu, Yizhen Wang, Feifan Zhou, Fuhong Cai

**Affiliations:** Sanya Research Institute, School of Biomedical Engineering, Hainan University, Sanya 572024, Chinazhouff@hainanu.edu.cn (F.Z.)

**Keywords:** phototherapy, light dose, wearable sensing system, temperature field distribution

## Abstract

Phototherapy is a physical treatment modality that utilizes natural or artificial light sources and harnesses radiant energy to treat diseases. Dynamic monitoring of the actual light dose received by tissues is crucial to the success of phototherapy. However, most current phototherapy devices feature bulky and complex hardware and depend on fixed parameters or surface measurements for dose estimation, failing to provide precise, real-time monitoring of light dose distribution that is tailored to individual users, specific treatment sessions, and different body regions. Furthermore, most of these devices are incapable of generating tunable and stable LED light. This study presents a preliminary diffusion equation-based proof-of-concept for a wearable, integrated dual-mode sensing system for real-time dynamic monitoring of tissue light dose and temperature change. The system, controlled by a single-chip microcontroller, rapidly extracts key tissue optical parameters via a custom multi-wavelength LED optical probe and provides real-time feedback on light dose distribution through a dynamic tissue optical simulation model. To expand the monitoring dimensions, the system innovatively integrates a thermal sensor. This sensor enables synchronous monitoring of the temperature field in the treatment area, thereby allowing for an estimation of the combined photothermal effect. The system features a compact design, user-friendly operation, fast and stable communication, and repeatable and reliable detection. With promising clinical application prospects, it holds the potential to evolve into a portable, home-use, safe, effective, wearable, and cost-effective phototherapy device.

## 1. Introduction

Precise dynamic monitoring of light dosage is essential for achieving safe and effective phototherapy. This is particularly critical in applications such as photodynamic therapy (PDT) and low-level light therapy, where appropriate dosing plays a decisive role in ensuring therapeutic efficacy and avoiding tissue damage [1]. However, due to the highly heterogeneous optical properties of biological tissues, traditional dose estimation methods that rely on fixed parameters exhibit significant limitations. They often fail to account for dynamic variations across different individuals, anatomical sites, and even throughout the treatment process [2]. This issue of “dosage uncertainty” has become a major bottleneck limiting the advancement of phototherapy toward greater precision. Consequently, the development of technologies capable of providing real-time feedback on the actual light dosage distribution within tissues has emerged as both a pressing need and a key challenge in driving the progress of precise phototherapy.

To address this challenge, the field of tissue optics provides the theoretical foundation. The modeling of light transport in tissues has evolved from the classical diffusion approximation theory [3] to Monte Carlo (MC) simulation [4], with the latter being regarded as the gold standard. The spatially resolved diffuse reflectance model established by Farrell et al. [5] laid the foundation for non-invasive measurement, while research by Kienle et al. [6] demonstrated that the diffusion approximation introduces significant errors when the source–detector distance approaches the transport mean free path. For layered tissues such as skin, the two-layer model theory developed by Takatani and Graham [7] confirmed the substantial contribution of deep tissues to the reflectance signal. However, high-accuracy models are often accompanied by high computational costs. In response, researchers have developed multiple strategies to balance precision and efficiency. For example, Alexandrakis et al. [8] proposed a hybrid MC-diffusion approximation model; Yudovsky and Pilon [9] established a fast semi-empirical analytical formula applicable to two-layer media based on extensive MC simulation data; and Fang [10] and Cai et al. [11] significantly accelerated MC simulations by leveraging GPU parallel computing technology and specific model optimization strategies, respectively, enabling their potential for real-time applications, including in areas such as skin characterization. The broadband optical property parameters of tissues systematically measured by Bashkatov and Tuchin [12,13], along with the inverse adding–doubling method proposed by Prahl [14], provided critical input for the construction and validation of these models.

Phototherapy hardware systems primarily utilize LEDs or laser diodes to generate a light source for irradiating lesion tissue [15,16]. Thanks to their compact structure and stable performance, LEDs have attracted significant attention in recent years and are now widely used as light sources in biosensing systems [17]. Multi-wavelength LED arrays have become the core light source choice for portable diffuse reflectance spectroscopy (DRS) systems due to their compact size, low-power consumption, and high stability [18,19]. Recently, research by Zhang et al. [20] further demonstrated that by optimizing the spectral design and mixing methods of multi-channel LEDs, target spectra can be effectively simulated with a limited number of hardware channels. This provides a new perspective for the design of compact systems. On the detection side, Hennessy et al. [21] systematically investigated the influence of probe geometry on sampling depth. Their conclusions provide crucial guidance for designing multi-distance probes with depth-resolving capability to extract layered tissue information. In more cutting-edge exploration, Lacerenza et al. [22] developed a wearable, wireless time-domain near-infrared spectroscopy system, enabling the monitoring of cerebral and muscular hemodynamics. This represents a significant milestone in the development of wearable optical devices. Meanwhile, Troy and Thennadil [23] further complemented the optical properties of skin in the long-wavelength near-infrared region, providing a basis for the design of broad-spectrum systems.

Despite significant progress, current technologies still face multiple bottlenecks in meeting the demand for real-time, in vivo clinical monitoring, with challenges being particularly pronounced in achieving dynamic closed-loop monitoring. First, synchronizing and monitoring the illumination and sensing elements in real time present certain challenges. In particular, light undergoes strong scattering [24] during propagation through biological tissues, requiring highly sensitive sensors to acquire optical signals from biological tissues for real-time monitoring [25]. Second, dynamic monitoring’s capability is insufficient. The majority of systems focus on static parameter measurements and lack schemes for the dynamic reconstruction and monitoring of light dosage, that is the cumulative effect of light field distribution over time. Third, monitoring dimensions are singular. Existing portable systems often involve trade-offs between performance and size and rarely integrate multimodal sensing. Biological effects in phototherapy arise from the synergistic action of optical and thermal energy [26]. Nevertheless, prevailing devices, including the latest therapeutic wearables, generally lack synchronous monitoring of the temperature field in the treatment area [27], which may lead to misinterpretation of therapeutic effects or thermal damage risks. Fourth, challenges exist regarding population specificity and practicality. Most optical model parameters are derived from data based on Western populations. As noted in a recent review, the representativeness of existing data on skin optical properties across different populations is limited, and consequently, systematic studies targeting the skin optical properties of Chinese populations are lacking [28]. Meanwhile, devices intended for real-world clinical application face the arduous task of reconciling the competing demands of miniaturization, low-power consumption, and high precision [29].

Focusing specifically on the discrete objective of dynamic light dosage monitoring, the limitations of existing technologies become even more pronounced. In terms of closed-loop monitoring capability, most research remains at the stage of parameter measurement or offline simulation, failing to effectively bridge real-time acquired parameters with dynamic dose calculation. In addition, most current portable monitoring devices focus exclusively on optical parameters, overlooking the simultaneous monitoring of the temperature field in the treatment area [27]. This shortfall cannot satisfy the clinical need for precise assessment of the combined photothermal effect. At the level of technological practicability, existing studies either prioritize algorithmic accuracy at the expense of real-time performance or achieve portability while struggling to ensure adequate sampling depth and signal-to-noise ratio [30]. Consequently, there remains a considerable gap to bridge before forming a stable and reliable “measurement–simulation–feedback” closed loop for clinical application.

In response to the aforementioned challenges, this study proposes an innovative “Measurement–Modeling–Monitoring” integrated solution, aiming to develop a wearable instrument integrated with dual optical/thermal sensing. The core of this solution is to construct a real-time feedback loop. A custom multi-wavelength LED optical probe rapidly extracts key tissue optical parameters (e.g., the effective attenuation coefficient). These parameters then directly drive an optimized, embedded numerical algorithm to dynamically reconstruct the internal three-dimensional light field distribution, thereby enabling continuous visual monitoring and early warning of light dose. This closed-loop design fundamentally distinguishes our approach from previous research where parameter measurement and dose calculation were disconnected [31]. The novelty of this work lies in three aspects. Methodologically, it establishes a closed-loop framework from limited-wavelength measurement to dynamic dose feedback, shifting from static parameter inversion to real-time dose monitoring. Systematically, it integrates optical and thermal sensors in a wearable probe, enabling the synchronous measurement of diffuse reflectance and temperature, which are used to estimate the effective attenuation coefficient, internal light fluence, and tissue temperature distribution via diffusion approximation and bioheat transfer theory. Technically, algorithmic optimization and compact design achieve balanced performance under wearable constraints. This integrated approach enables quantitative analysis of photothermal effects, providing an integrated platform for phototherapy monitoring research. It should be noted that this work primarily proposes the concept of a wearable dual-mode biosensing system and validates its performance in optical and temperature monitoring through preliminary experiments. In combination with a simplified diffusion-based theoretical model, initial calculations of light intensity and temperature distributions are also presented.

## 2. Materials and Methods

This study aims to develop a miniaturized, integrated illumination detection system incorporating both photoelectric and thermal sensors. To achieve dynamic monitoring of light dose, integrated hardware and algorithms are required. The hardware includes a light source and sensors. In this work, we introduce photodiodes and infrared sensors to simultaneously monitor both light intensity and temperature. For tissue optical modeling, the propagation of light in biological media is described by the radiative transfer equation.

Its time-domain integral-differential form is as follows:

$$\frac{1}{c(r)}\frac{\partial }{\partial t}L(r,s,t)+s\cdot 𝛻L(r,s,t)=-\mu_{t}(r)L(r,s,t)+\frac{\mu_{s}(r)}{4\pi }\int_{4\pi }p\left(s,s^{\prime }\right)L\left(r,s^{\prime },t\right)d\omega$$

where $c\left(r\right)$ is the speed of light in the medium; $\mu_{t}(r)$ is the total attenuation coefficient; $\mu_{s}(r)$ is the scattering coefficient; and $p\left(s,s^{\prime }\right)$ is the phase function.

In optical transmission of biological tissues, absorption coefficient and scattering coefficient are two key parameters. The differences in optical parameters among different individuals can be expressed through the above two parameters. The detection equipment applied to biological tissue optics requires the use of optical methods to calculate the above parameters. However, the calculation of the RTE equation mentioned above is very complex, and in practical applications, diffuse approximation is usually used for simplification. The specific analysis process is explained in Section 3.2. On the other hand, if the temperature distribution inside biological tissues is calculated, the classical heat conduction equation (PDE) can be used to describe the changes in heat inside biological tissues. The specific analysis of heat changes is explained in Section 3.4.

For the hardware modules, to provide sufficient data for optical computation of biological tissues, this work constructed a wearable light source and a photoelectric sensor module. The light source integrates a three-wavelength LED, providing more diverse phototherapy options. In addition, a thermal infrared sensor is integrated into the wearable light source module to acquire the surface temperature of the irradiated biological tissue in real time. A TSL element is selected as the photoelectric sensor in the wearable photoelectric sensor module. This sensor can acquire data and control exposure time via I2C. All modules have built-in Bluetooth modules, allowing for control via a mobile device app.

The modular design facilitates flexible spatial configuration of illumination and sensing. For example, for more robust calculation of biological tissue parameters, two or more sensors are typically required. We can place photoelectric sensor modules in different spatial locations to obtain spatially resolved optical signals, facilitating the calculation of biological tissue parameters. Simultaneously, the independent wearable light source module can also be deployed in a larger space to irradiate lesions over a large area.

**Figure 1:**
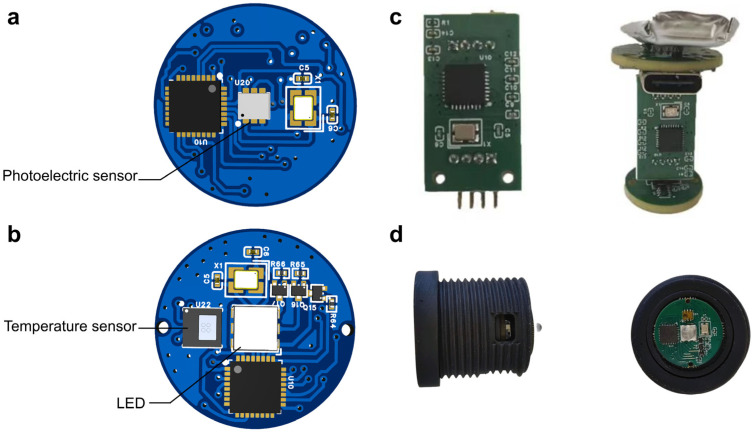
Structural design of the integrated illumination detection system architecture. (**a**) Front view of the printed circuit board (PCB) showing the photoelectric sensor and associated analog circuitry, responsible for detecting light dose distribution. (**b**) Front view of the printed circuit board (PCB) showing LED, the temperature sensor, and associated analog circuitry, responsible for detecting the temperature field distribution. (**c**) Actual device displaying various modules. (**d**) Physical prototype with enclosure.

The circuit and component designs for the photoelectric sensor module and the thermal sensor module are shown in Figure 1a and Figure 1b, respectively. A photograph of the fabricated circuit board is presented in Figure 1c, where the top layer is the driver module responsible for powering the system. As shown in Figure 1d, the circuit board is housed within a cylindrical black enclosure manufactured using 3D printing technology, thereby further enhancing the system’s esthetics and practicality.

### 2.1. Overall Architecture Based on ESP32-H2FH4

The ESP32-H2FH4 (Lexin, Shanghai, China) is a low-power microcontroller unit (MCU) system-on-a-chip (SoC) featuring 2.4 GHz Bluetooth Low Energy and 802.15.4 wireless communication capabilities. It integrates an RISC-V 32-bit processor, a Bluetooth Low Energy baseband, an 802.15.4 baseband, an RF module, and various peripheral interfaces. The I/O ports control MOSFETs to switch the LEDs on and off. Its built-in I2C interface is used to communicate with the photoelectric sensor and the thermal sensor, enabling high-speed data acquisition. The Bluetooth module supports wireless data transmission and can establish real-time communication with mobile devices. This modular design not only contributes to system miniaturization and compactness but also facilitates future functional expansion.

The LED we selected is a custom-designed 3-in-1 LED module, measuring 3.5 mm × 3.5 mm, with each LED chip inside being a 14-mil chip. Each LED chip has a maximum current of 50 mA and a voltage of 1.8 V. These three LED chips are connected in parallel in the circuit structure. The detection spectral range of TSL2591 (2.4 mm × 2 mm × 0.6 mm, ams OSRAM, Germany) is 450 nm–1000 nm. The noise floor under household white LED illumination is 4.98 µW/cm^2^. Under 850 nm LED illumination and 100 ms exposure time, its linear detection range is 0–250 µW/cm^2^. The thermal sensor utilized is the MLX90614 model (Melexis, Belgium), which offers a measurement accuracy of ±0.5 °C and features a response time of 0.5 s. The TSL2591 and MLX90614 are each connected to independent I2C buses, eliminating bus conflicts. However, achieving synchronized measurements requires attention to their different response times: the MLX90614 takes 0.5 s per reading, while the TSL2591 uses configurable integration times (typically 100–600 ms). In order to achieve synchronization, sensors are triggered simultaneously, and after each detection, the microcontroller has a sub-thread to read the corresponding detection data, ensuring that all measurements are synchronized to the same starting point.

This circuit module is powered by a 3.7 V LiPo battery with a 50 mAh capacity. Under normal operation, it achieves a Bluetooth latency of less than 50 ms. With this battery, the module supports approximately 0.5 h of continuous operation and maintains a standby time exceeding 24 h.

The system is housed in a 3D-printed black PLA enclosure featuring internal light baffles to prevent optical crosstalk and minimize ambient light interference. For thermal management, the LED operates at a maximum current of 50 mA, and the ESP32 microcontroller runs in low-power mode, resulting in minimal heat generation. Thermal compound is additionally applied to further enhance heat dissipation.

### 2.2. LED Module with Tunable Wavelength and Light Intensity

**Figure 2:**
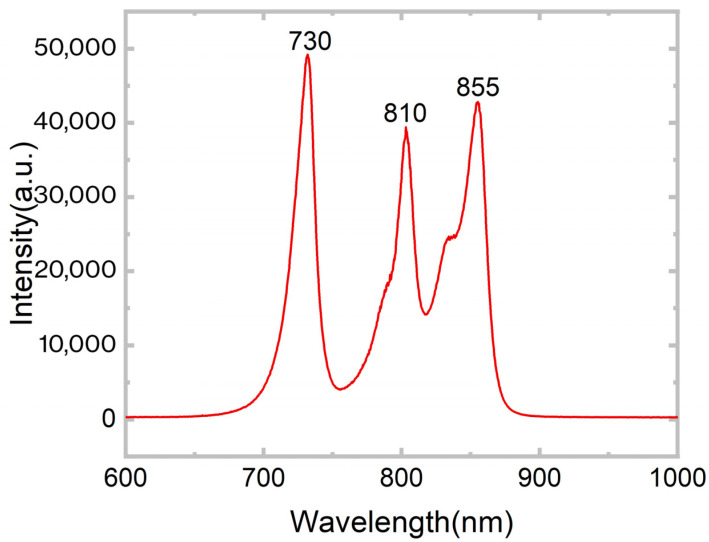
Spectral diagram corresponding to the light source. The three peaks correspond to the three LED wavelengths.

The excitation light source in our system comprises three LED chips with central wavelengths of 730 nm, 810 nm, and 855 nm (Figure 2). To address the insufficient output power from the I/O ports of the ESP32 microcontroller, a dedicated transistor amplification circuit was designed. This circuit effectively amplifies the current sourced from the control board, rendering it adequate to drive the LEDs and thereby achieve stable and uniform illumination. Regulation of the optical output power is accomplished by adjusting the resistance of the current-limiting resistor connected in series with each LED. This method offers ease of operation and facilitates continuous adjustment of the light intensity.

### 2.3. Advanced Detection Module with Integrated Dual Sensors

To ensure accurate measurement of both light dose and temperature distribution, the detection module of this system integrates a TSL2591 photoelectric sensor and a thermal sensor. The TSL2591 photoelectric sensor, serving as the core unit of the detection module, obtains the effective attenuation coefficient in real time by detecting the intensity of the emitted light, thereby enabling the deduction of light dose distribution. The thermal sensor is employed for real-time monitoring of temperature distribution.

The TSL2591 is a digital sensor with an internal ADC; after signal conversion by the internal ADC, it outputs digital signals directly to the microcontroller. The TSL2591 photodetector and MLX90614 infrared temperature sensor both communicate via I2C. The ESP32-H2 microcontroller reads digital data from both sensors, synchronizes the measurements via software timing, and transmits the data via BLE.

### 2.4. Closed-Loop Wireless Transmission Architecture

Building upon the integration of all modules, a closed-loop wireless transmission architecture was constructed utilizing the ESP32-H2FH4 chip. The photoelectric sensor and the thermal sensor collect light intensity and temperature data, respectively, and transmit these data to the chip. The chip then transmits the data to a mobile terminal via Bluetooth Low Energy (BLE) using its integrated low-power Bluetooth module. Upon receiving the data, the Android application not only supports real-time display and local storage but also enables remote adjustment of gain, exposure time, and control of LED switching. This closed-loop architecture enables human-device interactive feedback, making the system more suitable for wearable dynamic monitoring scenarios.

## 3. Results

### 3.1. Light Intensity Stability

**Figure 3:**
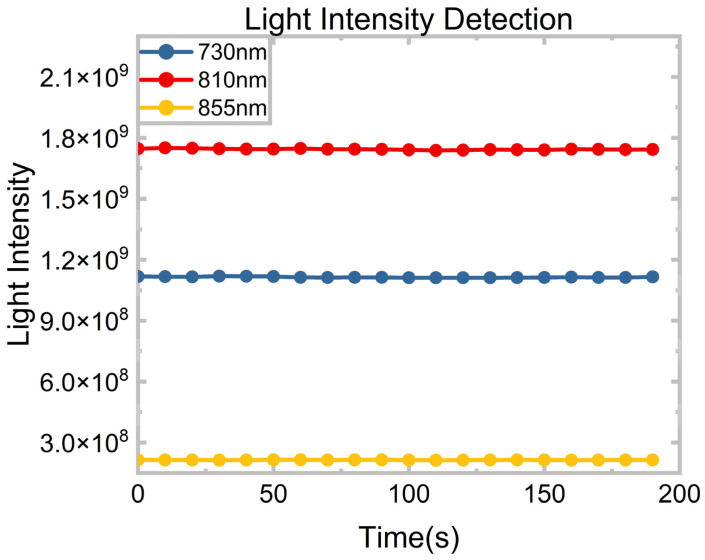
Light intensity stability analysis detection. Response of photodetectors to three wavelengths of LEDs. The plot shows minimal fluctuations in light intensity, confirming the stability of the light source.

To verify the stability of the LED light intensity, multiple measurements were performed on a fixed target using a photodetector. The system was placed flush against the subject’s skin surface, with the detector gain set to a moderate level and maintained at a 4 mm distance from the LED light source. For each measurement, only a single-wavelength LED was activated. Detector readings were recorded at 10 s intervals, resulting in a total of 20 datasets collected. Statistical analysis of the measurement results revealed that the relative standard deviations (RSDs) for the three LED wavelengths were all below 0.3%, with specific values of 0.23%, 0.18%, and 0.27%, respectively. The time-series plot indicates that the output data exhibit minor fluctuations, with no occurrence of sudden spikes, drops, or trend drift (Figure 3). The experiments confirmed that the light intensity output of each wavelength LED in the system exhibits high repeatability and stability, meeting the requirements for phototherapy applications.

### 3.2. Effective Attenuation Coefficient Measurement

Human tissue is a highly scattering medium, where the scattering coefficient is significantly greater than the absorption coefficient. When the distance from the detection point to the light source is sufficiently large, the diffusion approximation of the Boltzmann transport equation is generally valid for describing the propagation of near-infrared light within human tissue. This relationship can be approximately represented by the following equation:

$$\frac{1}{v}\cdot \frac{\partial \left(\varphi_{d}\left(r,t\right)\right)}{\partial t}-D\cdot \nabla^{2}\varphi_{d}\left(r,t\right)+\mu_{a}\cdot \varphi_{d}\left(r,t\right)=S(r,t)$$

where *v* denotes the speed of light propagation within the tissue, $\varphi_{d}\left(r,t\right)$ represents the diffuse photon fluence rate in the medium, *r* is the distance, *D* is the diffusion coefficient, and S(r,t) accounts for the contribution of the light source to the photon distribution in the medium.

Furthermore, the steady-state diffusion equation in a homogeneous, semi-infinite medium can be further approximated as follows:

$$R(r)\approx \frac{\sqrt{3}}{2\pi r^{2}}\left(1+\frac{2}{3}A\right)e^{-\mu_{eff}r}\sqrt{\frac{\mu_{a}}{\mu_{s}^{\prime }}}$$

where R(r) denotes the detected radiant flux at a distance r from a unit-intensity, steady-state source after light transmission through the tissue; A is a boundary correction factor related to internal reflection; $\mu_{a}$ is the absorption coefficient; $\mu_{s}^{\prime }$ is the reduced scattering coefficient; and $\mu_{eff}$ is the effective attenuation coefficient, defined as $\mu_{eff}=\sqrt{3\mu_{a}\mu_{s}^{\prime }}$. This is the most critical parameter under the diffusion approximation, as it determines the exponential attenuation rate of light in tissue.

To calculate the effective attenuation coefficient, it is necessary to know the radiant flux values at two points located at different distances from the light source. This relationship can be approximated as follows:

$$\left\{\begin{array}{c} R\left(r_{1}\right)=\frac{\sqrt{3}}{2\pi {r_{1}}^{2}}\left(1+\frac{2}{3}A\right)e^{-\mu_{eff}r_{1}}\sqrt{\frac{\mu_{a}}{\mu_{s}^{\prime }}}\\ R\left(r_{2}\right)=\frac{\sqrt{3}}{2\pi {r_{2}}^{2}}\left(1+\frac{2}{3}A\right)e^{-\mu_{eff}r_{2}}\sqrt{\frac{\mu_{a}}{\mu_{s}^{\prime }}} \end{array}\right.$$

Taking the ratio of the two equations above yields the following:

$$\frac{R\left(r_{1}\right)}{R\left(r_{2}\right)}=\frac{{r_{2}}^{2}}{{r_{1}}^{2}}e^{\mu_{eff}(r_{2}-r_{1})}$$

Therefore, the formula for solving the effective attenuation coefficient can be expressed as follows:

$$\mu_{eff}=\frac{\ln\frac{R\left(r_{1}\right){r_{1}}^{2}}{R\left(r_{2}\right){r_{2}}^{2}}}{r_{2}-r_{1}}$$

This formula indicates that calculating the effective attenuation coefficient requires only the ratio of the output data from two detectors and their respective distances from the light source.

This study recruited eight volunteers to measure the effective attenuation coefficient of the skin on the inner side of their forearms. The gender and skin color of the volunteers (according to the Monk Skin Tone (MST) Scale) were also listed in Table 1. All participants were in good health prior to testing, with no history of chronic diseases, skin conditions, or recent medication use. The skin areas examined were free of apparent wounds, scars, or abnormal pigmentation. For each volunteer, only a single-wavelength LED was activated during one measurement. The gain settings for the two detectors were identical, and their distances from the light source were fixed at 4 mm and 20 mm, respectively. By synchronously recording the output data from both detectors and applying the theoretical solution formula, the effective attenuation coefficient of the skin tissue at that wavelength was calculated. To minimize random errors, three repeated measurements were performed for each wavelength, and the average value was taken as the final result.

The effective attenuation coefficient of light in skin tissue exhibits inter-individual variation, and the attenuation characteristics of the same tissue also differ across wavelengths. This coefficient demonstrates good stability over short time periods. For instance, three consecutive measurements of the same wavelength performed on the same volunteer yielded results of 0.142 mm^−1^, 0.141 mm^−1^, and 0.143 mm^−1^, indicating excellent measurement repeatability.

The effective attenuation coefficient comprehensively reflects the combined absorption and scattering characteristics of biological tissue to light. It serves as a crucial parameter for predicting light penetration depth in tissue, planning phototherapy dosage, and optimizing treatment wavelengths, holding an indispensable position in biomedical optics for therapeutic applications. When measured using our system, the effective attenuation coefficient requires no introduction of excessive complex parameters; the calculation process is streamlined, and the results exhibit high accuracy and stability. This is of significant importance for the subsequent rapid feedback of the true internal light dose distribution and the realization of precise optical therapy.

### 3.3. Validation of Effectiveness

**Figure 4:**
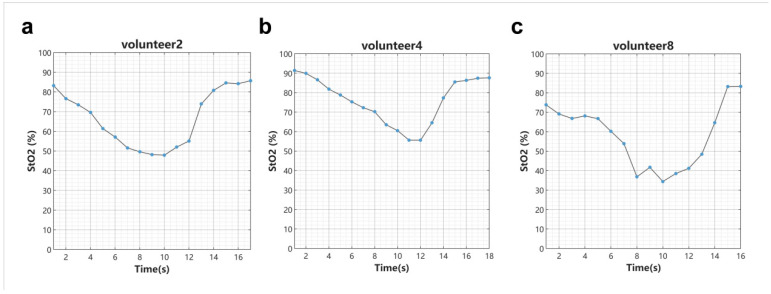
The detection system proposed in this work was used to monitor rSO_2_ in the arm muscles of three volunteers. At time T = 0, the muscle in the detection area transitions from a relaxed to a compressed state, and rSO_2_ gradually decreases. At approximately T = 10–12 s, the muscle begins to relax, and rSO_2_ gradually returns to baseline.

The detection probe was placed on the skin overlying the forearm muscle. A consequent decrease in regional oxygen saturation (rSO_2_) was observed during muscle contraction, followed by a recovery to baseline levels upon muscle relaxation. We use two LEDs, 730 nm and 855 nm, to sequentially irradiate the skin tissue, and use two sensors to detect the diffuse reflection intensity when the two LEDs are lit. For formula $R(r)\approx \frac{\sqrt{3}}{2\pi r^{2}}\left(1+\frac{2}{3}A\right)e^{-\mu_{eff}r}\sqrt{\frac{\mu_{a}}{\mu_{s}^{\prime }}}$, we define OD=−lgR(r). The above equation takes the derivative of the distance to obtain $\frac{\partial OD}{\partial r}=\frac{1}{\ln10}\left(\sqrt{3\mu_{a}\mu_{s}^{\prime }}+\frac{2}{r}\right)$. By changing the derivative dr to a difference, we can obtain $\frac{{\mu_{a}}^{\lambda_{1}}}{{\mu_{a}}^{\lambda_{2}}}={\left(\frac{{\Delta OD}^{\lambda_{1}}-\frac{2\cdot ∆r}{r\cdot \ln10}}{{\Delta OD}^{\lambda_{2}}-\frac{2\cdot ∆r}{r\cdot \ln10}}\right)}^{2}$. Here, ${\mu_{a}}^{\lambda_{1}}$ and ${\mu_{a}}^{\lambda_{2}}$ represent the absorption coefficients at wavelengths $\lambda_{1}$ and $\lambda_{2}$, respectively. Assuming that the absorption coefficient is mainly composed of oxygenated hemoglobin and reduced hemoglobin, regional oxygen saturation can be obtained from equation $rSO_{2}=\frac{{\varepsilon_{Hb}}^{\lambda_{1}}-{\varepsilon_{Hb}}^{\lambda_{2}}\cdot {\left(\frac{{\Delta OD}^{\lambda_{1}}-\frac{2\cdot ∆r}{r\cdot \ln10}}{{\Delta OD}^{\lambda_{2}}-\frac{2\cdot ∆r}{r\cdot \ln10}}\right)}^{2}}{{\left(\frac{{\Delta OD}^{\lambda_{1}}-\frac{2\cdot ∆r}{r\cdot \ln10}}{{\Delta OD}^{\lambda_{2}}-\frac{2\cdot ∆r}{r\cdot \ln10}}\right)}^{2}\cdot \left({\varepsilon_{HbO_{2}}}^{\lambda_{2}}-{\varepsilon_{Hb}}^{\lambda_{2}}\right)-\left({\varepsilon_{HbO_{2}}}^{\lambda_{1}}-{\varepsilon_{Hb}}^{\lambda_{1}}\right)}$. Here, $\varepsilon_{HbO_{2}}$ and $\varepsilon_{Hb}$ represent the Molar extinction coefficient of oxygenated hemoglobin and reduced hemoglobin. By using the above formula, the changes in rSO_2_ during muscle relaxation and tension can be monitored, as shown in Figure 4. At time T = 0, the muscle in the detection area transitions from a relaxed to a compressed state, and rSO_2_ gradually decreases. At approximately T = 10–12 s, the muscle begins to relax, and rSO_2_ gradually returns to baseline. This pattern is consistent with physiological characteristics.

The formula above is derived based on the simplified form of the diffusion equation in semi-infinite homogeneous tissue. This demonstrates that this simplified form can monitor the trend of rSO_2_ changes within localized regions of complex skin tissue. It indirectly indicates that using this simplified form allows for estimating the distribution of light intensity inside biological tissues with reasonable consistency under the conditions tested.

### 3.4. Internal Photon Fluence Rate Distribution

When $z_{0}\ll r$ and $z_{0}\ll z$, a simplified form of the internal photon fluence rate formula can be derived via Taylor expansion:

$$\Phi (r,z)\approx \frac{P}{4\pi D}\cdot \frac{2(z_{0}+2AD)z}{R^{3}}(1+\mu_{\mathrm{eff}}R)e^{-\mu_{\mathrm{eff}}R}$$

where P is the power of the isotropic point source, $D=\frac{1}{3(\mu_{a}+\mu_{s}^{\prime })}\approx \frac{1}{3\mu_{s}^{\prime }}$, $z_{0}=\frac{1}{\mu_{s}^{\prime }}$, r is the transverse distance, z is the depth, and $R=\sqrt{r^{2}+z^{2}}$.

By selecting a point $\left(r_{1},\;z_{1}\right)$ in the tissue as a fixed reference, the ratio of the photon fluence rate at any target measurement point $\left(r_{2},\;z_{2}\right)$ to that at the reference point is as follows:

$$\frac{\Phi_{2}(r_{2},z_{2})}{\Phi_{1}(r_{1},z_{1})}=\frac{z_{2}{R_{1}}^{3}}{z_{1}{R_{2}}^{3}}\left(\frac{1+\mu_{eff}R_{2}}{1+\mu_{eff}R_{1}}\right)e^{\mu_{eff}(R_{1}-R_{2})}$$

Based on the above formula, this study employs a relative measurement method to eliminate the dependency on absolute system parameters. Specifically, without requiring the specific values of the source power P or the tissue optical parameters D, A, and $z_{0}$, and by obtaining only the tissue’s effective attenuation coefficient $\mu_{eff}$ and the geometric coordinates of the measurement points, the photon fluence $\Phi_{2}$ at any internal tissue position can be calculated from the fluence $\Phi_{1}$ at a reference position.

**Figure 5:**
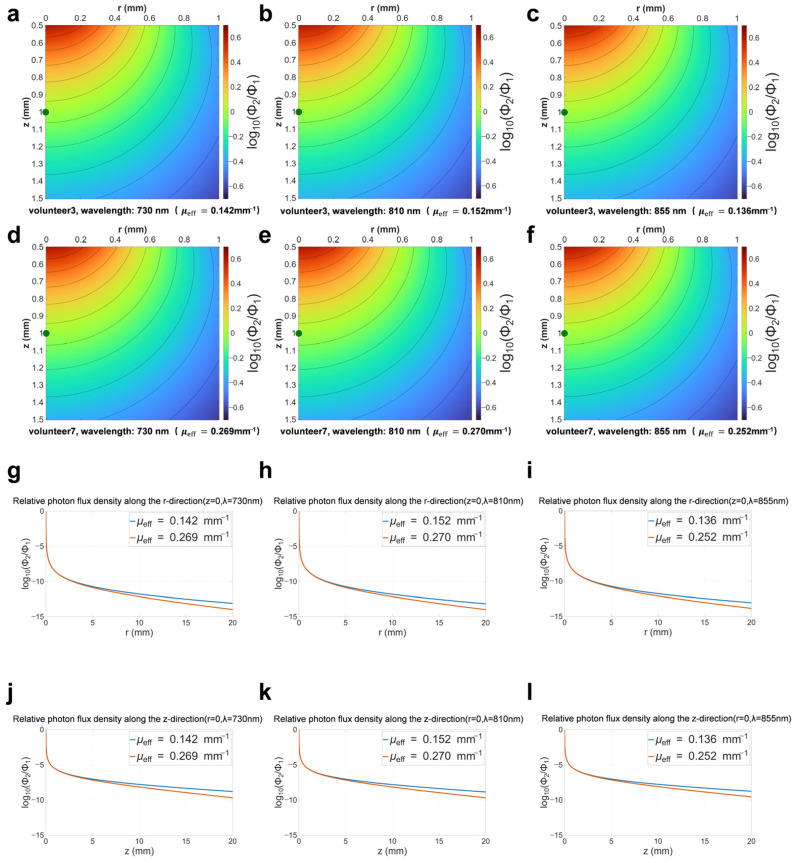
Relative photon flux density distribution on a logarithmic scale for a 1 mm × 1 mm area: (**a**) Volunteer 3, wavelength: 730 nm; (**b**) Volunteer 3, wavelength: 810 nm; (**c**) Volunteer 3, wavelength: 855 nm; (**d**) Volunteer 7, wavelength: 730 nm; (**e**) Volunteer 7, wavelength: 810 nm; (**f**) Volunteer 7, wavelength: 855 nm. (**g**–**i**) At z = 0, the normalized light intensity is a function of radial distance r; (**j**–**l**) at r = 0, the normalized light intensity is a function of depth z.

We selected the effective attenuation coefficients of Volunteer 3 and Volunteer 7 to generate the relative photon fluence rate distribution. As shown in Table 1, the effective attenuation coefficient of Volunteer 7 is approximately twice that of Volunteer 3. In terms of absolute values, the detected light intensity on the skin surface of Volunteer 7 is lower than that of Volunteer 3. However, here we are more concerned with the distribution of light intensity within the skin. Therefore, the light intensity distributions in Figure 5a–f are normalized. Visually, these intensity distributions are relatively similar. A shallow reference point directly beneath the LED light source is selected as $\left(r_{1}=0\;mm,\;z_{1}=1\;mm\right)$. In Figure 5g–i, we plotted the normalized curves showing how light intensity varies with r at the surface (z = 0). These three figures indicate that within the range of r = 0–5 mm, the intensity curves show a consistent trend. For the region where r > 5 mm, a larger effective attenuation coefficient leads to a more pronounced decrease in light intensity. A similar phenomenon can be observed in Figure 5j–l, which present normalized light intensity curves at different depths z, with r = 0. As the depth increases, a larger effective attenuation coefficient also results in a more significant attenuation of light intensity.

### 3.5. Temperature Distribution

**Figure 6:**
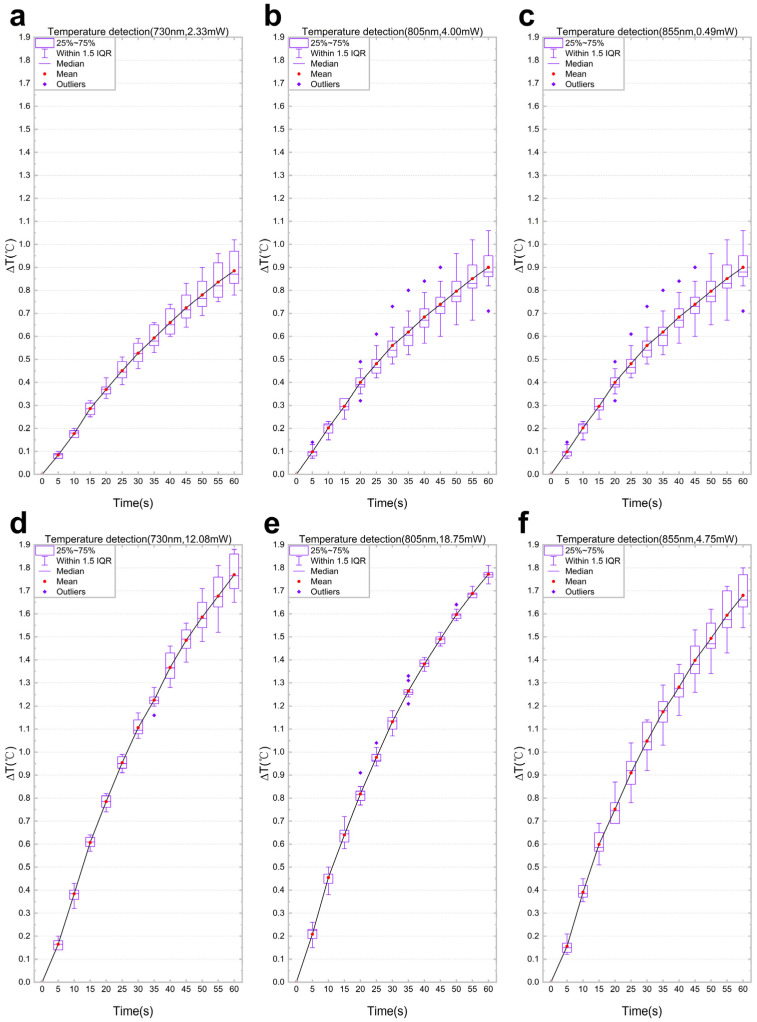
Box-and-whisker plot analysis of temperature rise over time recorded during 10 repeated irradiation experiments on the same target object at three wavelengths and two light power levels: (**a**) 730 nm, 2.33 mW; (**b**) 810 nm, 4.00 mW; (**c**) 855 nm, 0.49 mW; (**d**) 730 nm, 12.08 mW; (**e**) 810 nm, 18.75 mW; (**f**) 855 nm, 4.75 mW.

To evaluate the thermal effects of different optical powers on the target subject under various wavelengths, as well as the performance of the system, this study established two optical power levels (low-power group and high-power group) for each of the three wavelengths, resulting in a total of six experimental conditions. Under each condition, the same target subject was independently irradiated in 10 repeated experiments, with each irradiation lasting 60 s. Temperature data were collected at 5 s intervals using a temperature sensor to obtain the rise in the surface temperature of the target. Statistical analysis was performed on all repeated experimental data. The distribution characteristics of temperature rise at each time point were presented using box plots, with the mean values connected to illustrate the trend (Figure 6).

The experimental results show that under all six experimental conditions, the rise in the temperature of the target exhibited a monotonic increasing trend with prolonged irradiation time. Comparing the low-power and high-power groups under the same wavelength, it is evident that increasing the optical power significantly accelerates the rate of temperature rise. The narrow box widths, moderate whisker ranges, limited number of outliers, and minimal deviations in each group of box plots indicate that the temperature data from the 10 repeated experiments exhibit good consistency and repeatability. This verifies the stability of the experimental system and the reliability of the measurement results.

According to the Pennes bioheat equation, for a one-dimensional semi-infinite tissue Z≥0 with light incident on the surface, the steady-state temperature distribution satisfies the following equation:

$$k\frac{d^{2}T}{dz^{2}}+\mu_{a}\phi_{0}e^{-\mu_{eff}z}+\rho_{b}c_{b}w_{b}\left(T_{a}-T\right)=0$$

where k represents the tissue thermal conductivity; $\rho_{b}$, $c_{b}$, and $w_{b}$ are the blood density, specific heat capacity, and perfusion rate, respectively; and $T_{a}$ denotes the arterial blood temperature, which is typically equal to the core body temperature $T_{0}$. By setting $m^{2}=\frac{\rho_{b}c_{b}w_{b}}{k}$, solving the above equation, and applying the boundary conditions $T\left(0\right)=T_{s}$ (surface temperature) and $T\left(\infty \right)=T_{0}$, the analytical solution is obtained as follows:

$$T\left(z\right)=T_{0}+\left[u_{s}+\frac{\mu_{a}\phi_{0}}{k\left({u^{2}}_{eff}-m^{2}\right)}\right]e^{-mz}-\frac{\mu_{a}\phi_{0}}{k({u^{2}}_{eff}-m^{2})}e^{-\mu_{eff}z}$$

where $u_{s}=T_{s}-T_{0}$. In typical biological tissues, blood perfusion causes the temperature distribution to attenuate from the surface toward the interior, approaching $T_{0}$. Moreover, with $\mu_{eff}\gg m$, the influence of internal heat generation becomes relatively minor. Consequently, the temperature distribution is primarily governed by the surface temperature and heat dissipation via blood perfusion and can be approximated as follows:

$$T_{z}\approx T_{0}+\left(T_{s}-T_{0}\right)e^{-mz}$$

where the typical value of *m* is 0.0876 mm^−1^, *T*_0_ is typically taken as 36.5 °C, and *T_s_* can be obtained in real time from a temperature sensor placed in close contact with the skin.

**Figure 7:**
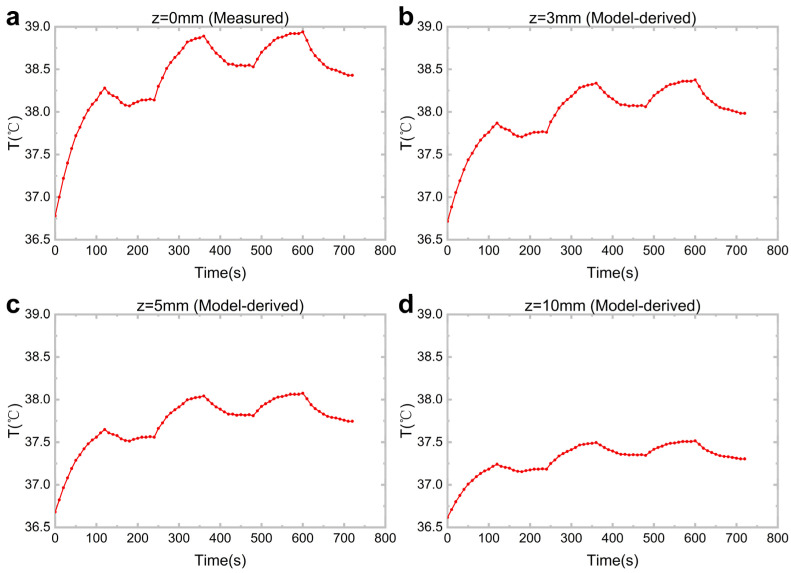
Temperature oscillations at different tissue depths under periodic LED illumination: (**a**) z = 0 mm, surface temperature directly measured by a temperature sensor; (**b**) z = 3 mm, theoretically predicted temperature profile; (**c**) z = 5 mm, theoretically predicted temperature profile; (**d**) z = 10 mm, theoretically predicted temperature profile.

The system was placed in close contact with the skin surface, and the LED light source was operated according to a fixed timing sequence: alternating between 2 min on and 2 min off, completing three full on/off cycles, with a total measurement duration of 6 min. The acquired surface temperature time-series data $T_{s}(t)$ were substituted into the approximate formula above, enabling the calculation of temperature fluctuations at various tissue depths over time (Figure 7).

## 4. Discussion and Conclusion

In this study, we demonstrate the feasibility of integrating a dual-mode sensor in a wearable system for real-time dynamic light dosimetry in tissues. The system integrates optical and thermal sensing within a compact, low-power wearable device, enabling simultaneous monitoring of light dose distribution and temperature changes during phototherapy. Through hardware design, algorithmic optimization, and experimental validation, preliminary results indicate the system’s capability to provide stable light output, extract key tissue optical parameters (such as the effective attenuation coefficient), reconstruct internal photon fluence distributions, and monitor temperature variations with depth and time.

This study employs a homogeneous semi-infinite diffusion approximation model as a computationally efficient preliminary solution for real-time wearable light dosimetry. We fully acknowledge the inherent limitations of this model when applied to complex biological tissues such as skin. Skin is a typical layered medium, and its optical properties (e.g., absorption and scattering coefficients) vary significantly across different layers, including the epidermis, dermis, and subcutaneous tissue. Notably, melanin content in the epidermis plays a dominant role in light absorption; thus, the current model cannot fully overcome the influence of skin pigmentation (i.e., variations in melanin concentration) on the accuracy of light dose reconstruction. Given these limitations, the proposed framework is more suitable for revealing relative trends in light distribution within tissues rather than providing absolute quantitative measurements.

Moreover, the temperature modeling presented in this study assumes a simplified one-dimensional semi-infinite medium with homogeneous perfusion. In reality, tissue thermal dynamics involve complex three-dimensional heat diffusion, heterogeneous blood perfusion, and intricate boundary conditions. The approximation used in this work, while analytically tractable, may not fully capture the actual temperature distribution in heterogeneous tissues or near anatomical boundaries. Future work should incorporate more sophisticated bioheat transfer models to improve thermal prediction accuracy. Meanwhile, in the optothermal calculation model, we employed a constant-temperature boundary condition without accounting for the impact of blood flow on temperature changes. For wearable systems, the hardware is closely connected to the skin, minimizing the influence of ambient temperature on the skin. Thus, under the premise of simplified calculations, a constant-temperature boundary condition can be adopted. However, blood flow within the skin is ubiquitous, and neglecting it would result in overestimated predictions of internal tissue temperatures.

Additionally, the system’s operational wavelength range (730–855 nm) inherently limits penetration depth, confining sensing primarily to superficial tissue layers. This restricts the accuracy of derived optical parameters and dose distributions for deeper target tissues. Future iterations could incorporate longer near-infrared wavelengths to enable depth-resolved characterization. The distance between the light source and the two sensors is 4.2 mm and 20.1 mm, respectively. According to the literature [32], the reduced scattering coefficients of skin at 725 nm, 800 nm, and 850 nm are 1.41 mm^−1^, 1.23 mm^−1^, and 1.13 mm^−1^, respectively. Assuming an absorption coefficient of 0.01 mm^−1^ (which is much smaller than the reduced scattering coefficient), the corresponding transport mean free paths (MTF) are 0.704 mm, 0.806 mm, and 0.877 mm, respectively. The distance from the first sensor to the light source is 4.2 mm. When expressed in terms of MTF, this distance corresponds to 6.0 MTF, 5.2 MTF, and 4.78 MTF for the three wavelengths, respectively. Classical theory requires the distance to be greater than 5 MTF for the diffusion approximation to be valid. Despite the fact that for 850 nm the 4.2 mm distance between the first sensor and the light source is slightly less than the 5 MTF requirement, it remains within the theoretical boundary, and the resulting error is expected to be minor. In future work, we will optimize the hardware configuration by increasing the distance between the first sensor and the light source by 0.2 mm, ensuring that it meets the 5 MTF criterion.

Nevertheless, several limitations should also be acknowledged. First, the current system employs a simplified diffusion model and a limited set of three wavelengths (730, 810, and 855 nm), which may not fully capture the complexity of heterogeneous or multi-layered tissues, especially for chromophores with absorption peaks outside this range. Future work could incorporate broader spectral coverage (e.g., 600–1000 nm) and layered tissue models to improve accuracy. Second, while the system demonstrates promising performance in controlled settings with eight volunteers, clinical validation across a larger and more diverse patient population (e.g., number > 20) and under various treatment conditions is necessary to establish its robustness, generalizability, and clinical thresholds for dose safety. Third, further miniaturization and power optimization are needed to enhance wearability and enable long-term monitoring. The current prototype’s battery life under continuous operation is approximately 0.5 h; future iterations should aim for 0.5 h to support typical treatment sessions.

In conclusion, this work demonstrates the feasibility of a wearable dual-mode sensing approach for personalized phototherapy monitoring. The system ability to concurrently measure light dose and temperature offers a more integrated monitoring profile compared to systems that monitor only optical parameters. A key aspect of this work is the integration of measurement, modeling, and monitoring within a single wearable device. Unlike conventional systems that rely on pre-determined optical parameters or offline simulations, our approach utilizes a custom multi-wavelength LED probe to acquire tissue-specific optical properties. These properties then serve as inputs to a simplified diffusion model (semi-infinite homogeneous medium) to estimate light distribution within the tissue. This analytical approximation was chosen because it enables rapid real-time calculation of the effective attenuation coefficient and internal photon fluence distribution, which is essential for wearable devices with limited computational resources.

Furthermore, the integration of a thermal sensor addresses an important consideration in phototherapy monitoring. Photothermal effects are inseparable from therapeutic outcomes and safety, yet temperature monitoring is not always included in wearable systems. Our system provides synchronous optical and thermal data, allowing for the simultaneous observation of light exposure and surface temperature changes. For patient safety during extended use, the sensor provides continuous temperature monitoring to detect abnormal rises; if the temperature exceeds a safety threshold, the system can automatically shut off the light source, preventing potential thermal damage. In the future, we will recruit more volunteers to conduct clinical trials. At the same time, this work will also combine more accurate models, such as Monte Carlo simulation for analyzing the distribution of light and heat inside biological tissues [33], to verify and optimize the numerical calculation model of this work.

## Figures and Tables

**Table 1:** Effective attenuation coefficient measurement (mm^−1^).

Volunteer	730 nm_1	730 nm_2	730 nm_3	730 nm_Mean	810 nm_1	810 nm_2	810 nm_3	810 nm_Mean	855 nm_1	855 nm_2	855 nm_3	855 nm_Mean	MST	Sex
1	0.142	0.141	0.144	0.142	0.153	0.156	0.156	0.155	0.139	0.136	0.139	0.138	3	Female
2	0.148	0.149	0.149	0.149	0.166	0.165	0.164	0.165	0.146	0.145	0.146	0.146	3	Female
3	0.141	0.141	0.143	0.142	0.151	0.151	0.153	0.152	0.134	0.138	0.135	0.136	3	Female
4	0.150	0.149	0.151	0.150	0.169	0.169	0.167	0.168	0.145	0.144	0.147	0.145	3	Male
5	0.145	0.145	0.144	0.145	0.155	0.154	0.154	0.154	0.138	0.137	0.135	0.137	3	Female
6	0.213	0.213	0.214	0.213	0.223	0.227	0.224	0.225	0.208	0.209	0.207	0.208	4	Male
7	0.262	0.260	0.259	0.269	0.272	0.269	0.268	0.270	0.253	0.251	0.251	0.252	4	Male
8	0.249	0.239	0.241	0.243	0.256	0.250	0.251	0.252	0.234	0.231	0.230	0.232	4	Male

## Data Availability

The original contributions presented in this study are included in the article. Further inquiries can be directed to the corresponding author.
